# Transient R-wave amplitude attenuation owing to air entrapment during extravascular implantable cardioverter-defibrillator implantation: Delayed reassessment as a potential bail-out strategy

**DOI:** 10.1016/j.hrcr.2025.10.045

**Published:** 2025-11-08

**Authors:** Tomonobu Yanase, Satoshi Oka, Kohei Ishibashi, Nobuhiko Ueda, Mitsuru Wada, Kengo Kusano

**Affiliations:** Department of Cardiovascular Medicine, National Cerebral and Cardiovascular Center, Suita, Japan

**Keywords:** Air entrapment, Defibrillation threshold, Extravascular implantable cardioverter-defibrillator, Substernal lead, Ventricular arrhythmia

**Key Teaching Points**

• Air entrapment in the substernal space may transiently reduce R-wave sensing during extravascular implantable cardioverter-defibrillator implantation.
• In this setting, R-wave sensing and defibrillation testing failure may resolve spontaneously as the entrapped air dissipates over time.
• In borderline cases, delayed reassessment, rather than immediate system revision, may be a feasible and appropriate strategy when air entrapment is involved.

## Introduction

The extravascular implantable cardioverter-defibrillator (EV-ICD) offers ventricular tachycardia termination by delivering antitachycardia pacing followed by defibrillation therapy, without the risks of transvenous systems.^1^^,^^2^ In the previous studies, EV-ICD implantation was unsuccessful in 3.1%–5.4% of patients despite attempted implantation, primarily owing to inadequate R-wave amplitude sensing and failed defibrillation threshold (DFT) testing.^1^^,^^3^ Even among successful implantations, intraoperative system repositioning was necessary in at least 20% of cases.^4^ Preoperative prediction of EV-ICD sensing and defibrillation performance remains challenging, given its strong dependence on individual thoracic anatomy and the positions of both the substernal lead and the generator. Furthermore, other factors—such as air entrapment surrounding the substernal lead and its ring electrodes—may influence EV-ICD sensing and defibrillation performance, although their impact and solutions remain unclear.^5^ Herein, we report a case of transient R-wave amplitude attenuation during EV-ICD implantation, likely caused by air entrapment around the substernal lead.

## Case report

A 73-year-old man (height 156 cm; body weight 65 kg) with cardiac sarcoidosis and reduced left ventricular ejection fraction was indicated for primary prevention implantable cardioverter-defibrillator (ICD) therapy after documented nonsustained monomorphic ventricular tachycardia. No atrioventricular block was present. Given these clinical characteristics, he underwent implantation of an EV-ICD (Aurora, Medtronic, Inc.).

**Figure 1:**
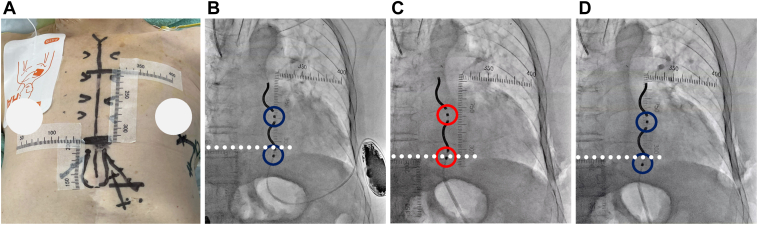
Intraoperative fluoroscopy. **A:** The top of the cardiac silhouette, xiphisternal junction, and both parasternal borders were visualized using radiopaque tape on the skin surface. Anteroposterior fluoroscopic views show **(B)** the initial substernal lead position with the ring 1 and ring 2 electrodes highlighted (*blue circles*), **(C)** the substernal lead repositioned slightly rightward (*red circles*), and **(D)** the final position, where the R-wave amplitude remained below the success criteria despite securing the ring electrodes being near the initial position (*blue circles*). Ring 2 was located inferior to the level of the xiphisternal junction (*white dashed line*).

**Figure 2:**
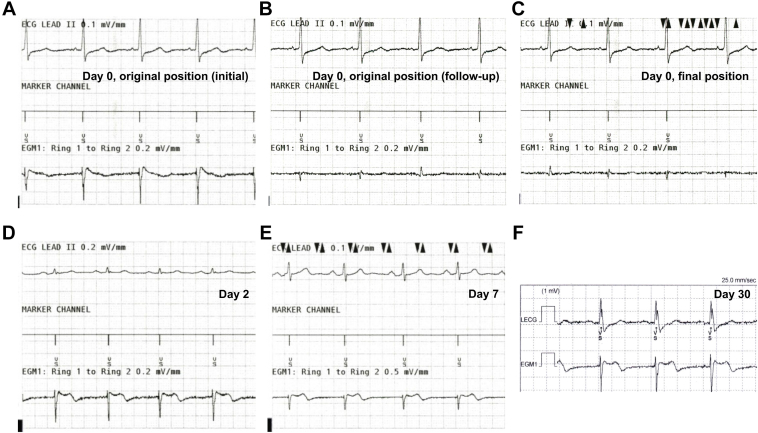
Device electrogram on the ring 1–ring 2 electrodes. **A:** At the initial sensing test, the device electrogram demonstrated an adequate R-wave amplitude on the ring 1–ring 2 vector (1.5–2.5 mV) without P-wave or myopotential oversensing. **B:** During implantation, the R-wave amplitude declined. **C:** At the final position, the R-wave amplitude was 0.6 mV despite the ring electrode replacement near the initial position. **D:** On postoperative day 2, the R-wave amplitude improved to 1.6 mV, meeting the success criteria. **E:** By postoperative day 7, amplitude further improved to 2.4 mV. **F:** At 1-month follow-up, the R-wave amplitude was 2.6 mV, with no inappropriate therapies observed.

The procedure was performed under general anesthesia with mechanical ventilation. The substernal lead was tunneled through the standard left-sided substernal space under the guidance of key anatomic landmark visualization using radiopaque tape, as we previously reported (Figure 1A).^6^ The initial R-wave amplitude on the ring 1–ring 2 electrodes vector was 1.5–2.5 mV without P-wave or myopotential oversensing (Figure 2A), meeting the success criteria: (1) R-wave amplitude of >1 mV with no P-wave amplitude of >0.2 mV or (2) R-wave amplitude of at least 10 times greater than the detected P-wave amplitude (0.2–0.3 mV). The generator was implanted in the preoperatively planned position, determined by the left-most and posterior wall silhouettes of the left ventricle in the anterior-posterior and left lateral views, respectively. However, initial DFT testing failed to terminate induced ventricular fibrillation (VF) with a 15 J shock, even when followed by a 30 J shock, necessitating external defibrillation.

**Figure 3:**
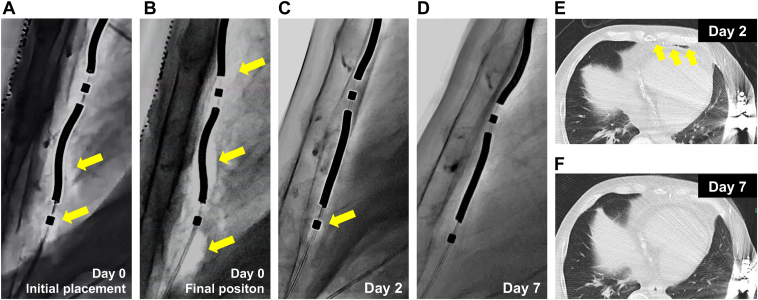
Resolution of air entrapment around the substernal lead. Serial lateral fluoroscopic views show **(A)** at the initial position, **(B)** at the end of the index operation, **(C)** on postoperative day 2, and **(D)** 1 week after implantation. Entrapped air surrounding the ring 1 and ring 2 electrodes progressively decreased over time and resolved completely by 1 week. Radiolucent areas indicating entrapped air are highlighted with *yellow arrows*. Computed tomography **(E)** on postoperative day 2 demonstrated residual entrapped air surrounding the substernal lead, **(F)** whereas repeat imaging on postoperative day 7 confirmed complete resolution of the air entrapment.

As a potential cause of DFT failure, fluoroscopic review suggested that the device generator was positioned relatively anterior to the cardiac silhouette line of the left ventricular posterior wall. To optimize the shock vector, the generator was repositioned dorsally toward the posterior ventricular silhouette; during this maneuver, the R-wave amplitude on the ring 1–ring 2 vector was attenuated (Figure 2B) despite no apparent change in the lead position. At this point, air around the ring 2 electrode was already evident (Figure 3A; Supplemental Video) and was retrospectively considered a possible cause of the reduced R-wave amplitude. Concerned about potential slight substernal lead migration, we retunneled and replaced the lead through a similar left-sided substernal course to the original position. The newly obtained R-wave amplitude did not meet the sensing threshold (<1.0 mV) on the ring 1–ring 2 vector, even near a previous successful site and slightly more rightward positioning (Figure 1C).

After multiple retunneling procedures, we observed a progressive accumulation of air surrounding the substernal lead, particularly adjacent to the ring 2 electrode in a fluoroscopy (Figure 3B; Supplemental Video). Despite attempts to mitigate the issue—including Trendelenburg positioning, Valsalva maneuver, and carbon dioxide insufflation over the wound site—the sensing did not improve. Given the previously confirmed adequate sensing and procedural stability, the substernal lead was ultimately secured in its original position under radiopaque tape guidance (Figure 1D). Given that there were no theoretical concerns regarding the lead or generator positioning, the implantation procedure was completed despite the R-wave amplitude remaining low (0.6 mV) (Figure 2C), and defibrillation efficacy was not confirmed at that time. Thus, defibrillation therapy of the EV-ICD was withheld, pending reassessment.

On postoperative day 2, device interrogation showed spontaneous improvement in R-wave amplitude to 1.6 mV (Figure 2D), and DFT testing was successful at 30 J. Fluoroscopy and computed tomography demonstrated that the entrapped air around the substernal lead had decreased (Figure 3C and 3E) (Supplemental Video).

1 week after implantation, the R-wave amplitude further improved to 2.4 mV (Figure 2E), with fluoroscopy demonstrating complete resolution of the air around the substernal lead (Figure 3D; Supplemental Video), also confirmed by computed tomography (Figure 3F). On repeat DFT testing, induced VF was successfully terminated with a 10 J safety margin. The patient was discharged the next day. At 1-month follow-up, the R-wave amplitude was 2.6 mV (Figure 2F), with no complications or inappropriate therapies observed.

## Discussion

This case highlights 2 clinically relevant issues in EV-ICD implantation: (1) sensing and defibrillation performance can only be assessed intraoperatively using the ring 1–ring 2 sensing vector and DFT testing, and (2) transient attenuation of R-wave amplitude may occur owing to entrapped air surrounding the substernal lead, making accurate intraoperative sensing assessment challenging.

Although the sensing ability of subcutaneous ICD (S-ICD) can be assessed both preoperatively and intraoperatively with an optimal sensing vector automatically selected between a ring electrode and generator (or between ring electrodes) based on R- and T-wave amplitudes after implantation,^7^ EV-ICD sensing can only be assessed intraoperatively using the ring 1–ring 2 vector. Consequently, sensing performance depends on the relationship between the substernal lead’s ring electrodes and the surrounding environment.^8^ Air, a poor conductor, may insulate electrodes from cardiac signals and current flow.^9^

In this patient, extensive substernal air accumulation likely caused a reduction in R-wave amplitude. Given the spontaneous improvement of R-wave amplitude over 48 hours in the absence of lead migration, air entrapment was the most plausible explanation. Air entrapment is a recognized phenomenon in the early phase after nontransvenous lead implantation, potentially interfering with accurate R-wave detection. A previous study reported that 1.2% of S-ICD recipients experienced device malfunction owing to air entrapment, expressed as artifacts, baseline drift, and reduced R-wave amplitude.^9^ One of the most frequent complications is inappropriate shock delivery. With respect to EV-ICD, only 1 case report of substernal air entrapment has been published to date, where ventricular oversensing caused by artifacts on the ring 1–ring 2 vector occurred 3 hours after implantation and resolved by the next day.^5^ To the best of our knowledge, this is the first report demonstrating improvement in R-wave detection in parallel with resolution of substernal air entrapment, supporting the feasibility of delayed reassessment of sensing and defibrillation performance after EV-ICD implantation.

Notably, entrapped air around the ring 2 electrode was already evident at initial placement (Figure 3A) and may have contributed to early attenuation of the R-wave amplitude. During substernal access, blunt finger dissection through diaphragmatic attachments for EV-ICD lead placement, inadvertent introduction of air into the substernal tunnel may not be completely avoidable. Taking this into consideration, positioning ring 2 near the diaphragmatic entry site may increase the risk of air entrapment. According to the results of a simulation study, positioning ring 2 more than 1 cm above the xiphisternal junction is recommended for optimal defibrillation performance.^10^ Furthermore, the recommended ring 2 position is also optimal for preventing R-wave amplitude attenuation caused by entrapped air. However, in patients with a small body habitus, the limited distance between the xiphisternal junction and the top of the cardiac silhouette provides only a narrow margin compared with the distance between the substernal lead tip and ring 2 (approximately 90 mm). In the present case, the ring 2 was positioned inferior to the xiphisternal junction (Figure 1) and close to the diaphragmatic entry site, which exposed the ring 2 to entrapped air. This observation suggests that patients with a smaller body size may be at higher risk, although further investigation is warranted.

Unlike S-ICD, surface maneuvers such as saline injection into the lead tunnel or skin massage are not feasible in EV-ICD implantation. In our institution, we routinely use strategies to facilitate air elimination, including Valsalva maneuver on mechanical ventilation and carbon dioxide insufflation around the wound site. However, in this case, prevention and reduction of air entrapment could not be achieved despite using these methods in the Trendelenburg position. Repeated substernal retunneling may predispose to air entrapment and R-wave amplitude attenuation. Notably, because adequate sensing had initially been confirmed, a delayed reassessment strategy was adopted, ultimately allowing spontaneous resolution of the entrapped air and restoration of acceptable device parameters. This case demonstrates that a delayed reassessment strategy may be appropriate when implantation criteria are initially met, potentially avoiding unnecessary system revision and complications such as organ injury from multiple retunneling procedures and infection from prolonged procedure time. Radiopaque tape guidance proved valuable not only for safe tunneling and repeated sensing tests^6^ but also for repositioning the substernal lead to its original location.

Because adequate R-wave amplitude could not be obtained intraoperatively and undersensing during induced VF was a concern, DFT testing was performed on days 2 and 7 after implantation. Therefore, it cannot be determined whether the subsequently successful defibrillation efficacy was caused solely by EV-ICD repositioning or absorption of the entrapped air.

In a systematic review, 94.8% of S-ICD malfunctions potentially attributable to air entrapment were recorded within the first week after implantation.^9^ Consistent with this, the entrapped air surrounding the substernal lead in our case completely resolved within 1 week. Although further investigation is needed to determine the optimal observation period before considering system replacement or conversion to a transvenous ICD, 1 week may be sufficient for reassessment of EV-ICD sensing and defibrillation performance.

### Limitations

There are several limitations to this reassessment strategy after EV-ICD implantation. First, even when an adequate R-wave amplitude is initially achieved, once retunneling is performed, reproducing the exact original trajectory is practically impossible. EV-ICD sensing and defibrillation parameters can be sensitive to minor changes in lead path or depth. Second, air entrapment around a substernal lead is not always demonstrable on fluoroscopy. In S-ICD, Ali et al^9^ reported that radiologic confirmation of air entrapment was achieved in only 27.7% of device malfunctions potentially attributable to air. They further suggested that postimplant air entrapment is likely under-recognized and may represent a bystander condition^9^; a similar phenomenon may apply to EV-ICD. Finally, in many cases, because the contribution of air entrapment to suboptimal parameters cannot be determined until postimplant reassessment, the procedure may need to be completed while leaving open the possibility of reoperation—including lead repositioning or conversion to another system. Given that EV-ICD implantation is performed under general anesthesia, this contingency should be carefully addressed in pre- and postoperative informed consent.

## Conclusion

Air entrapment after EV-ICD implantation may transiently reduce R-wave amplitude. In cases where initial sensing was acceptable, a delayed reassessment strategy may allow time for spontaneous resolution and confirmation of successful implantation.

## Disclosures

Dr Kengo Kusano has received honoraria from Daiichi Sankyo and Medtronic Japan and research grants from Medtronic Japan, Abbott Medical Japan, Boston Scientific Japan, Biotronik Japan, GE Precision Healthcare LLC, Johnson & Johnson, and JSR, outside the submitted work. Drs Kohei Ishibashi, Nobuhiko Ueda, and Satoshi Oka have received remuneration for lectures from Medtronic Japan, Inc.
