# Supplementary material for: Transient R-wave amplitude attenuation owing to air entrapment during extravascular implantable cardioverter-defibrillator implantation: Delayed reassessment as a potential bail-out strategy
Source: HeartRhythm Case Rep. 2025 Nov 8;12(2):214–8. doi: 10.1016/j.hrcr.2025.10.045 (PMC12922527; doi:10.1016/j.hrcr.2025.10.045)
Supplement: Supplementary Video [file mmc1.docx]

**Supplementary video**

**Resolution of air entrapment around the substernal lead**

Serial lateral fluoroscopic views during the implantation, on postoperative day 2, and one week after implantation are demonstrated. Entrapped air surrounding the ring 1 and ring 2 electrodes progressively decreased over time and resolved completely by one week. Radiolucent areas indicating entrapped air are highlighted with red circles.
